# Functional brain plasticity during L1 training on complex sentences: Changes in gamma‐band oscillatory activity

**DOI:** 10.1002/hbm.25470

**Published:** 2021-05-04

**Authors:** Peng Wang, Thomas R. Knösche, Luyao Chen, Jens Brauer, Angela D. Friederici, Burkhard Maess

**Affiliations:** ^1^ Max Planck Institute for Human Cognitive and Brain Sciences Brain Networks Group Leipzig Germany; ^2^ Beijing Normal University College of Chinese Language and Culture Beijing; ^3^ Max Planck Institute for Human Cognitive and Brain Sciences Department of Neuropsychology Leipzig Germany; ^4^ Friedrich Schiller University Office of the Vice‐President for Young Researchers Jena Germany

**Keywords:** brain plasticity, first language (L1) training, language network, magnetoencephalography, oscillatory brain activity, speech processing

## Abstract

The adult human brain remains plastic even after puberty. However, whether first language (L1) training in adults can alter the language network is yet largely unknown. Thus, we conducted a longitudinal training experiment on syntactically complex German sentence comprehension. Sentence complexity was varied by the depth of the center embedded relative clauses (i.e., single or double embedded). Comprehension was tested after each sentence with a question on the thematic role assignment. Thirty adult, native German speakers were recruited for 4 days of training. Magnetoencephalography (MEG) data were recorded and subjected to spectral power analysis covering the classical frequency bands (i.e., theta, alpha, beta, low gamma, and gamma). Normalized spectral power, time‐locked to the final closure of the relative clause, was subjected to a two‐factor analysis (“sentence complexity” and “training days”). Results showed that for the more complex sentences, the interaction of sentence complexity and training days was observed in Brodmann area 44 (BA 44) as a decrease of gamma power with training. Moreover, in the gamma band (55–95 Hz) functional connectivity between BA 44 and other brain regions such as the inferior frontal sulcus and the inferior parietal cortex were correlated with behavioral performance increase due to training. These results show that even for native speakers, complex L1 sentence training improves language performance and alters neural activities of the left hemispheric language network. Training strengthens the use of the dorsal processing stream with working‐memory‐related brain regions for syntactically complex sentences, thereby demonstrating the brain's functional plasticity for L1 training.

## Graphical abstract

We demonstrate brain plasiticity to language training with a longitudinal auditory experiment during which native German speakers were trained to understand syntactically complex sentences. Based on MEG recordings, we identified a gamma power decrease in the left BA 44 for the more complex syntactic structures but not for the simpler ones. Over the training days, a correlation was observed between performance and gamma‐ based connectivity for pairwise connections from and to BA 44.

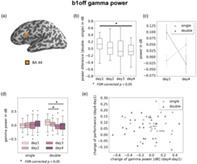

## 1 INTRODUCTION

Our brains are highly plastic in nature, which is fundamental for the optimization and adaptation of cognitive functions in response to everyday life challenges. The language domain, one of the most sophisticated cognitive domains in humans, provides an excellent window for studying this neurobiological property. In adults the language network is fully mature, both structurally and functionally (e.g., Skeide & Friederici, 2016). Nevertheless, it appears to remain plastic during the whole lifespan, as we continue to learn new words, adapt the use of known words and phrases to current conventions, and learn new languages. This plasticity is of special importance in the case of disease or injury. For example, clinical stroke studies have reported that damaged language networks could gradually reorganize within the spared language regions and perilesional tissues in the left hemisphere as well as newly recruited regions in the right hemisphere (Brownsett et al., 2014; Hartwigsen & Saur, 2019). Evidence for the ongoing plasticity of the intact language systems mainly comes from adult second language (L2) learning studies. They suggest that even short‐term learning experience can induce detectable functional changes in language‐related brain regions, such as the left inferior frontal gyrus (IFG) and the superior temporal gyrus (STG) – for a review, see Tables 1 and 2 in Li, Legault, & Litcofsky, 2014. Moreover, studies with different types of grammar have revealed that training over hours or days leads to significant functional changes in Broca's area and its dorsal connection to the left temporal lobe, that is, the arcuate fasciculus (Bahlmann, Schubotz, & Friederici, 2008; Bahlmann, Schubotz, Mueller, Koester, & Friederici, 2009; Flöel, de Vries, Scholz, Breitenstein, & Johansen‐Berg, 2009; Friederici, Bahlmann, Heim, Schubotz, & Anwander, 2006; Uddén, Ingvar, Hagoort, & Petersson, 2017).

However, it is unknown whether and to what extent the language network is modulated, if healthy participants undergo comprehension training with syntactically challenging sentences in their mother tongue. Therefore, we conducted a longitudinal experiment with adult, native German speakers. On four consecutive days, the participants listened to complex German sentences and were required to answer probing questions that tested their understanding of the thematic role assignment indicating who is doing what to whom. The sentences contained either single or double embedded relative clauses. An analysis of seven European languages including German showed that in modern spoken languages, sentences containing multiple center embedded relative clauses are quite sparsely used, and that even in writing, the maximal level of center embedding is limited to three (Karlsson, 2007). Therefore, these sentences were thought to be ideal material for the current experiment.

We expected functional changes over training days within the language network, especially in the core language areas, such as Brodmann's area (BA) 44, a key syntactic region, and the posterior superior temporal gyrus/sulcus (pSTG/pSTS), which subserves syntactic and semantic information integration (Brauer, Anwander, & Friederici, 2011; Friederici, 2017a; Friederici, 2017b; Friederici et al., 2006; Wilson et al., 2011; Zaccarella, Schell, & Friederici, 2017). Furthermore, language‐related working memory systems, such as the left inferior frontal sulcus (IFS) and the left inferior parietal cortex (IPC), might also need to be functionally modulated to successfully comprehend complex sentences (Gruber & von Cramon, 2003; Makuuchi, Bahlmann, Anwander, & Friederici, 2009; Makuuchi & Friederici, 2013).

During training, brain activity was recorded with magnetoencephalography (MEG), which monitors tiny magnetic field variations caused by synchronous synaptic activity in neuronal populations. The high temporal resolution of this method allowed us to focus precisely on the final closures of the hierarchically center‐embedded structures, the stage in sentence processing at which all embedded structures are integrated into a consistent meaning. Because local information processing in the brain, as well as interactions between areas, are often characterized by frequency specific activity (brain oscillations or rhythms) (see below), we performed source localized time‐frequency analysis on the acquired MEG data. Accumulated evidence assigns brain rhythms in different frequency bands to various processes related to the language domain (for reviews, Bastiaansen & Hagoort, 2006; Maguire & Abel, 2013; Meyer, 2018; Murphy & Benítez‐Burraco, 2019; Prystauka & Lewis, 2019; Martorell, Morucci, Mancini, & Molinaro, 2020). For instance, the gamma band (>30 Hz) has been reported to reflect syntactic structure building (Ding, Melloni, Zhang, Tian, & Poeppel, 2016; Nelson et al., 2017) or, more generally, sentence‐level information composition (see Martorell et al., 2020 for a recent review). Recent intercranial measurements in language related regions of the left hemisphere (Nelson et al., 2017) showed that high gamma power gradually increases when reading sentences word by word, until it suddenly drops when the words can be merged into a phrase (phrase closure). This increasing gamma power might be interpreted as a signature of syntactic structure building or as reflecting increasing working memory demands. Consequently, the decrease at the phrase boundary could be related either to the completion of the syntactic structure or to the sudden release of working memory (Lundqvist, Herman, Warden, Brincat, & Miller, 2018). In the current study, we would therefore expect a gamma power increase over the course of the sentence and a drop at the final embedding position. This should be more pronounced for sentences with double embedding. This effect should also be localized in certain language‐specific areas as hypothesized above. The alpha (8–12 Hz), beta (13–30 Hz), and theta (4–7 Hz) bands are assumed to reflect cognitive demands such as working memory (Bonhage, Meyer, Gruber, Friederici, & Mueller, 2017; Jensen & Tesche, 2002; Onton, Delorme, & Makeig, 2005; Proskovec, Wiesman, Heinrichs‐Graham, et al., 2018; Prystauka & Lewis, 2019; Weiss & Mueller, 2012). Yet, these effects are less consistent during sentence processing (for reviews, Weiss & Mueller, 2012; Prystauka & Lewis, 2019). As a result, we included the following five frequency bands in our analyses: alpha (8–12 Hz), beta (13–30 Hz), and theta (4–7 Hz), which are supposed to reflect working memory processes, and high/low gamma (55–95 Hz, 31–45 Hz), which are related to syntactic structure building (also involving syntactical working memory).

In summary, we sought to characterize functional brain plasticity of healthy participants during L1 training of complex syntax processing. Specifically, we investigated the following questions. (1) Can we identify a sentence‐complexity‐dependent training effect in behavior? (2) In which frequency bands and in which parts of the extended neural language network is this sentence‐complexity‐dependent training effect observed, with regard to within‐area and inter‐area synchronization? (3) Which interconnections between regions, measured as performance change, are related to training success? Answers to these questions may be useful to constrain mechanistic models of information processing in the brain (Kunze, Haueisen, & Knösche, 2019; Schmidt, Hahn, Deco, & Knösche, 2020; Wang & Knösche, 2013), which in turn are expected to deliver deeper insights into how aspects of language processing are implemented in the brain.

## 2 METHODS

### 2.1 Participants

Thirty, native German speakers (15 females) participated in this study. The mean age was 27 years, ranging from 20 to 34. All participants were right‐handed according to the Edinburgh handedness test: mean 89, range from 40 to 100 (Oldfield, 1971). The mean reading span was 3.7 with a standard deviation of 0.9. Participants reported no neurological diseases or hearing impairment and were naïve to the purposes of the experiment. Participants gave written informed consent prior to the experiment and were paid 9 Euros per hour for participation. The study was approved by ethics committee of the University of Leipzig.

### 2.2 Stimulus materials and procedure

**FIGURE 1:**
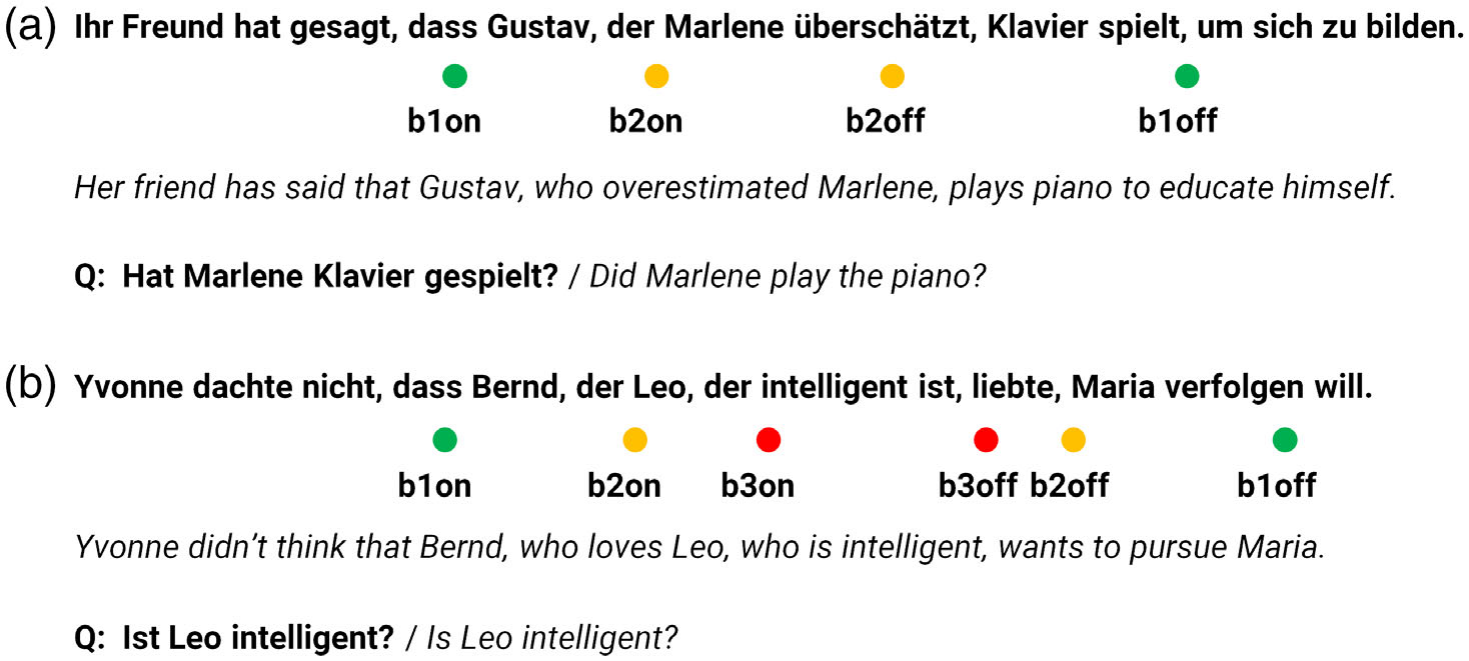
Examples of presented sentences in German (English translations in italics): (a) with single and (b) with double center embedding. We used the data segment starting at *b1on* as a reference to compute the relative spectral power change for all other marked time points. The marker *b1on* represented the beginning of the relative clause containing all hierarchical embedding. Q: Probing questions for the presented examples

Participants were presented with two types of German sentences with single (Figure 1(a)) and double hierarchical center embedding (Figure 1(b)). All sentences started with an introductory phrase followed by a relative clause. For each sentence, nouns and verbs were randomly drawn from a pool, ensuring that there were no semantic cues for thematic role assignment. The same noun or verb was not permitted to reappear in the sequence of presentation within the following five stimuli, irrespective of condition. In order to avoid surprising or implausible semantic relationships or unknown vocabulary, the material was rated in a pre‐experiment by 21 raters on a Likert scale ranging from 1‐to‐6 along the following dimensions: vocabulary comprehensibility (very incomprehensible to very comprehensible), emotionality of content (emotionally very unexciting to emotionally very exciting), and semantic plausibility (very implausible to very plausible). Sentences were removed from the material if the ratings were beyond pre‐defined thresholds. Sentences were required to be comprehensible (score of 3.5 or higher), rather nonemotional (score of 3.5 or lower), and semantically plausible (score of 3.5 or higher). The beginning of each relative clause (brace, marking the boundary of embedding) was marked with *bxon* while the final verb of it was marked with *bxoff*. The *x* was *1*, *2*, or *3* for double embedding sentences, and *1* or *2* for single embedding sentences.

The experiment included four sessions carried out on four of the five working days (Monday–Friday) within 1 week. Stimulus presentation was controlled by the software “Presentation” (www.neurobs.com). Participants listened to 33 single and 33 double embedded sentences each day. In total, each participant listened to 264 different sentences during the 4 days. The 132 sentences per condition were drawn without repetition from a reservoir of 140 single embedded and 190 double embedded sentences. Each participant received an individual randomization of 264 sentences. None of the sentences were presented twice to the same participant. All sentences were spoken at a natural speed. Sentence duration varied between 3.0 and 10.5 s. Mean (±SD) sentence length was 6.3 s (±1.1) for the double embedded and 4.5 s (±0.8) for the single embedded sentences. Mean length of the first embedding (b1off – b1on) was 3.8 s (±0.7) for the double embedding and 2.1 s (±0.5) for the single embedding sentences. Each trial began with the participant pressing a button, after which a fixation star was presented. After 500 ms, the auditory presentation of a sentence started. The fixation star was replaced by a fixation cross 1,000 ms after the sentence ending. A content question was then presented auditorily, probing the understanding of the thematic role assignments. Thereafter, the button assignment was displayed on the screen for a maximum of 3,000 ms or until the participant responded. It consisted of two pictures presented side‐by‐side: one showed a green circle with a white check mark (“yes”), the other a red circle with a white cross (“no”). For each answer, participants received feedback (correct, incorrect, or too slow). In the case of an incorrect answer, the same sentence was repeated and additionally displayed on the screen and thereafter a second content question was asked. This question was also followed by the button‐assignment screen and the corresponding feedback to the participant. The next trial was started by a button press (see above). Overall, the average block length was less than 7 min. MEG data analysis and behavioral analysis were restricted to the first, auditory only, presentation of the sentences.

### 2.3 Language processing‐related regions of interest (ROIs) definition

**FIGURE 2:**
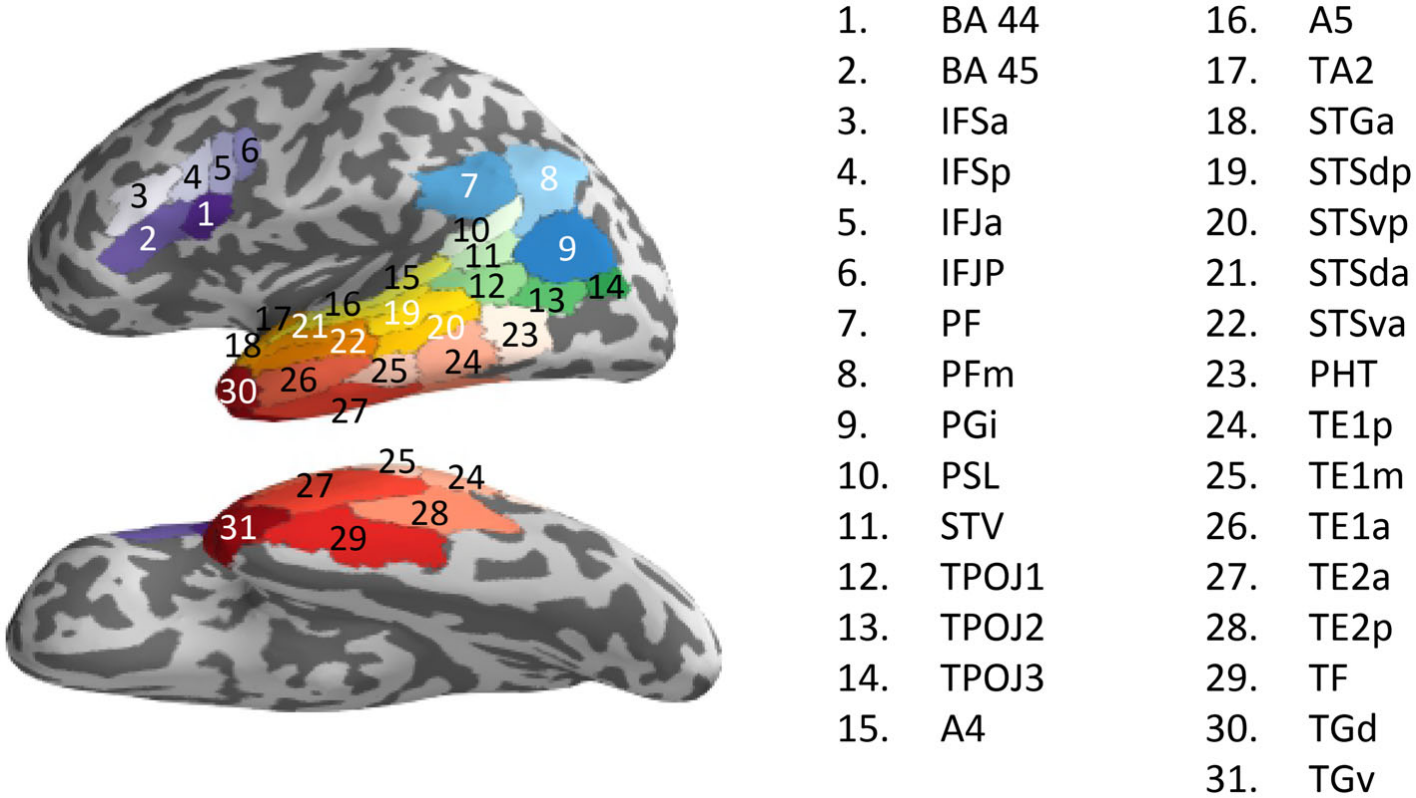
Regions of interest (ROI) comprising the language system in the left hemispheric cortex: Broca's area (BA 44, BA 45), inferior frontal gyrus (IFSa, IFSp, IFJa, IFJP), inferior parietal gyrus (PF, PFm, PGi), tempo‐pro‐parieto‐occipital junction (STV, PSL, TPOJ1‐3), superior temporal gyrus (TA2, STSGa, A4, A5), superior temporal sulcus (STSda, STSva, STSdp, STSdv), middle temporal gyrus (TE1a, TE1m, TE1p, PHT), inferior temporal gyrus (TE2a, TE2p, TF) and temporal pole (TGd, TGv). ROIs were labeled according to Glasser et al., 2016

We employed a surface‐based cortical brain atlas published recently by the human connectome project (Glasser et al., 2016) using the individual cortex surfaces as segmented and labeled by Freesurfer 6.0.0. Thirty‐one language‐relevant regions (ROIs) were selected from the complete set of 180 left hemispheric ROIs of the atlas (Figure 2), comprising the core left language network. Among the selected ROIs were regions important for processing hierarchical embeddings, such as BA 44 and the pSTG/pSTS (Friederici, 2017a, Friederici, 2017b), regions relevant for the verbal working memory system, such as the IPC (Gruber & von Cramon, 2003), and regions relevant for the syntactic working memory system, such as the IFS (Makuuchi et al., 2009; Makuuchi & Friederici, 2013).

### 2.4 Behavioral data analysis

Accuracy and reaction time of the participants' responses to the first question were analyzed.

### 2.5 MEG data acquisition and analyses

#### 2.5.1 MEG data acquisition, preprocessing, and power analysis

During the training sessions, we recorded MEG with a 306‐channel Elekta Neuromag Vectorview device at a sampling rate of 1,000 Hz and within a frequency band from DC to 330 Hz. Subjects were seated under the MEG helmet with their heads positioned as much inside in the helmet as possible. The raw data was first spatially filtered using signal space separation (SSS) via maxfilter© v2.2.15 utilizing spherical functions up to eleventh order for the head field model and up to the second order for the environmental field model. The SSS‐filter was applied to suppress environmental interferences, to interpolate the manually identified broken channels, and to transform all data of 1 day to the head position at the beginning of the first block of that day. Further data analysis was conducted using the MNE‐Python package Version 0.16 (Gramfort et al., 2013, 2014). The raw data were filtered offline with a 120 Hz low‐pass and a 0.3 Hz high‐pass filter. Both filters were single pass FIR type, utilizing a Hann‐Window and a transition band of 10 Hz or 0.3 Hz, respectively. In the next step, we used independent component analysis (ICA) to remove eye‐blink and heartbeat artifacts. Thereafter, we defined epochs of 0.5 s length starting with the event triggers at *b1on*, *b1off*, *b2on*, *b2off*, *b3on*, and *b3off* (see Figure 1). The length of the analysis window was defined by the minimum distance between the *b1off* position and the end of the sentences (*seoff*). The mean values over the entire epochs were subtracted to remove the DC background. Artifact rejection led to an average dropout rate of 0.6% (max: 6%) over all participants and days. The number of trials stayed the same between conditions (median difference: 0/95th‐percentil: ±1.

Within this paper, we decided to exclusively report the estimated brain activity and not to conduct sensor space analysis. This was because our hypotheses were related to brain regions and not to groups of sensors. For the localization, we constructed individual, single shell volume conductor and source models based on the individual T1‐weighted MR data. To this end, we utilized Freesurfer 6.0.0 for segmenting the inner skull surface and the cortical surface. Finally, Freesurfer also labeled the cortical surface according to Glasser et al. (2016), introducing the regions of interest we refer to below. We used the LCMV beamformer (Van Veen, Van Drongelen, Yuchtman, & Suzuki, 1997) to compute the source activity on the brain surface. The reconstructed current density was restricted to being perpendicular to the cortical surface, and was described by a single activity time course per spatial location. As a noise covariance matrix we utilized the mean noise covariance from the empty room measurements obtained before and after each recording session. In contrast, the data covariance matrices were computed separately for each day as common filters for all conditions and time points based on the whole sentence data. The power spectrum density (PSD) for each source was estimated using the multi‐taper method (4 Hz multi‐taper windowing, data zero‐padding to 2 s), separately for each subject, ROI, sentence type, and day as an average over all presented sentences. In a next step the 1/f pink noise background was estimated and subtracted from each average PSD separately. Total spectral powers for theta (4–7 Hz), alpha (8–12 Hz), beta (13–30 Hz), low gamma (31–45 Hz), and gamma (55–95 Hz) bands were estimated by averaging all frequency bins within the corresponding spectral window. Finally, relative power values were calculated by normalizing to the respective *b1on* power value separately for each subject, ROI, sentence type, and day. Hence, all subsequently reported spectral power values are relative powers.

#### 2.5.2 Functional connectivity analyses

For the connectivity analysis, we focused on the time point *b1off* (Figure 1) and bidirectional interactions between BA 44 and a set of 16 target ROIs from the inferior frontal cortex following the arcuate fasciculus towards the temporo‐parietal cortex (Friederici, 2011; Makuuchi & Friederici, 2013). Epochs were defined as 0–0.55 s relative to *b1off*, and padded to −0.3 – 1.0 s with recorded data to avoid edge effects. The data were downsampled to 500 Hz and projected into target brain regions using the LCMV beamformer (see above). For each source inside a target ROI, we computed the time‐frequency representation (TFR) using Morlet‐wavelet transform and averaged them to obtain the TFR of the entire ROI. For each time point of the TFR, 1/f pink noise background was estimated and subtracted.

We used 250 samples (time window from 0 to 0.5 s) to compute the Granger influence (see below) between pairs of ROIs. Consequently, we obtained 16 Granger influences per training day and sentence type. Additionally, we computed the ImCoh in the gamma spectral window of 55–95 Hz, for each source pair between two selected ROIs. Values of all included frequency bins and sources within the two ROIs were averaged. Since the ImCoh is either positive (phase difference between 0 and π) or negative (phase difference between ‐π and 0), we averaged the positive and negative values separately. Thus, for each pair of target ROIs, we had two phase‐phase synchrony values, reflecting either phase lead and phase lag of the first ROI compared to the second. Hence, we obtained 16 positive and 16 negative ImCoh values for each day and each sentence type, representing the phase‐coupling relationship between BA 44 and the other 16 dorsal pathway ROIs. We display positive ImCoh values if BA 44 is leading other ROIs and negative ImCoh values if BA 44 is lagging behind other ROIs. We decided to compute both Granger influence and ImCoh values because the two measures differ in the type of coupling they reflect. Imaginary coherence indicates phase‐phase coupling (or phase synchrony), which arises from rather tight coupling between the circuits. In contrast, the Granger influence measures amplitude‐amplitude coupling, arising from a looser coupling between the circuits. The two methods also differ in how they suppress trivial connectivity due to volume conduction. Imaginary coherence fully excludes phase angles close to zero, while this is not the case for the Granger influence. See below for more details.

##### Granger influence as connectivity estimate

Granger “causality” analysis (Geweke, 1982; Granger, 1969) is an asymmetric measure of the coupling relationship between two time series. The central idea behind this is the notion that if the prediction of one time series could be improved by using the knowledge of a second one, then the second time series is said to have a causal influence on the first (Wiener, 1956). Later, Wiener's idea was formalized in the context of linear regression models (Geweke, 1982; Granger, 1969). Briefly, we compare the prediction errors (also called residuals or innovations) of two autoregressive models of the current value of a time series *X(t)*: Model 1 using only samples of the past of *X(t)*, and Model 2 additionally taking into account the past of the second signal *Y(t)*. If the additional information from *Y(t)* significantly reduces the prediction error, then it is said that *Y(t)* is *Granger causal* of *X(t)*. If ∑_1_ and ∑_2_ are the variances of the errors of Models 1 and 2, respectively, then the Granger causality is:

$$F\left(Y\to X\right)=\ln\frac{\sum_{1}}{\sum_{2}}$$

Likewise, we can also compute the reverse Granger causality *F(X → Y)* and their difference Δ*F* (Roebroeck et al., 2005; Kayser et al., 2009):

$$\Delta F\left(X,Y\right)=F\left(X\to Y\right)-F\left(Y\to X\right),$$

which measures the major influence direction, concerning which time series has more influence over the other one. In this paper we refer to this term as *Granger Influence*.

The best model order *n* (i.e., how many past samples of the respective signals are used in the models), which represents the required amount of knowledge of past history, can be estimated through the Bayesian information criterion (BIC) when estimating the prediction error of the multivariate auto‐regressive model.

##### Imaginary coherence as connectivity estimate

Imaginary coherence (ImCoh; Nolte et al., 2004) measures the time‐lagged phase synchrony between two time series. It is the imaginary part of the complex‐valued coherency, which represents the normalized cross‐spectrum of two time series in frequency. By neglecting the real part, the measure avoids bias due to volume conduction effects (Nolte et al., 2004), but may also miss a true interaction if it is not time lagged. While the absolute value of ImCoh between two signals *X(t)* and of *Y(t)* is symmetric, its sign is reversed, indicating which of the two signals is leading and which is lagging.

#### 2.5.3 Statistical tests

We conducted a two‐factor analysis of the spectral power at the closure of the outer embedding (*b1off*) in all selected ROIs and frequency bands. The factors were *sentence complexity* (two levels: single or double embedding) and *training day* (four levels: day1–day4). We tested three hypotheses:

H0_1_: The power difference due to *sentence complexity* is independent of changes due to *training days* (interaction effect).

H0_2_: Syntactic complexity has no influence on the spectral power at *b1off* (*sentence complexity* effect).

H0_3_: Training has no influence on the spectral power at *b1off (training day* effect).

We used nonparametric tests since power, accuracy, and correlation are not normally distributed. Specifically, we applied the Wilcoxon signed‐rank method if not stated otherwise. For each selected ROI and frequency band, we performed three tests for H0_1_ (day1 vs. day2, day1 vs. day3, day1 vs. day4), one test for H0_2_ (double vs. single), and three test for H0_3_ (day1 vs. day2, day1 vs. day3, day1 vs. day4). To account for multiple testing (7 tests × 31 ROIs × 5 power bands = 108 tests), we applied false‐discovery‐rate‐correction (FDR‐correction, Benjamini & Hochberg, 1995) with a significance level of 0.05.

Subsequently, we computed the Spearman's correlation between the Granger influence change and the performance change for all 16 BA 44‐related ROI pairs, and reported the FDR‐corrected p‐values. The auto‐regressive model and the multivariate auto‐regressive model were computed using the Statsmodels (v0.11) software.

Additionally, we computed the Spearman's correlation between ImCoh change and performance change for all 16 BA 44‐related ROI pairs and both directions from BA 44 (positive ImCoh values) and to BA 44 (negative ImCoh values), and reported FDR‐corrected *p*‐values.

## 3 RESULTS

All reported effects have FDR‐corrected *p*‐values below 0.05.

### 3.1 Behavioral data analysis results

**FIGURE 3:**
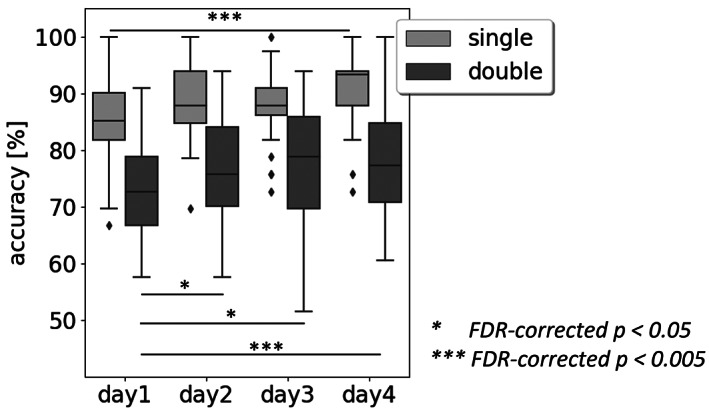
Participants' performance during the four training days. Accuracy of single embedded sentences significantly increased from day1 to day4. Accuracy of double embedded sentences increased significantly for days 2, 3, and 4 compared to day 1. The boxes show the interquartile range that stretches from the first quartile (25th percentile) to the third quartile (75th percentile) with the black line marking the median (50th percentile). The maximal whisker range is 1.5 times the interquartile range. Note, that the displayed whisker length depends on values within whisker range. Diamonds represent outliers, that is, values outside the whisker range

Considering the sentences with single embedding alone, accuracy was generally higher: 85%, 88%, 88%, and 93% (day1 to day4, see Figure 3). A significant change was only detected when comparing day1 with day4 (pairwise Wilcoxon signed‐rank test). Considering sentences with double embedding only, accuracy was lower at: 73%, 76%, 79%, and 77% (day1 to day4, Figure 3). Significant differences were observed for all days in comparison to day1 (pairwise Wilcoxon signed‐rank test). Moreover, the participants always scored higher for single compared to double embedded sentences on each day. Even on the last day, accuracy for double embedding was still lower than for single embedding on the first day (five pairwise Wilcoxon signed‐rank tests for single versus double embedding on each day as well as single embedding on day1 versus double embedding on day4, FDR‐corrected *p*‐values <0.005).

### 3.2 Spectral power as function of training days and sentence complexity

**FIGURE 4:**
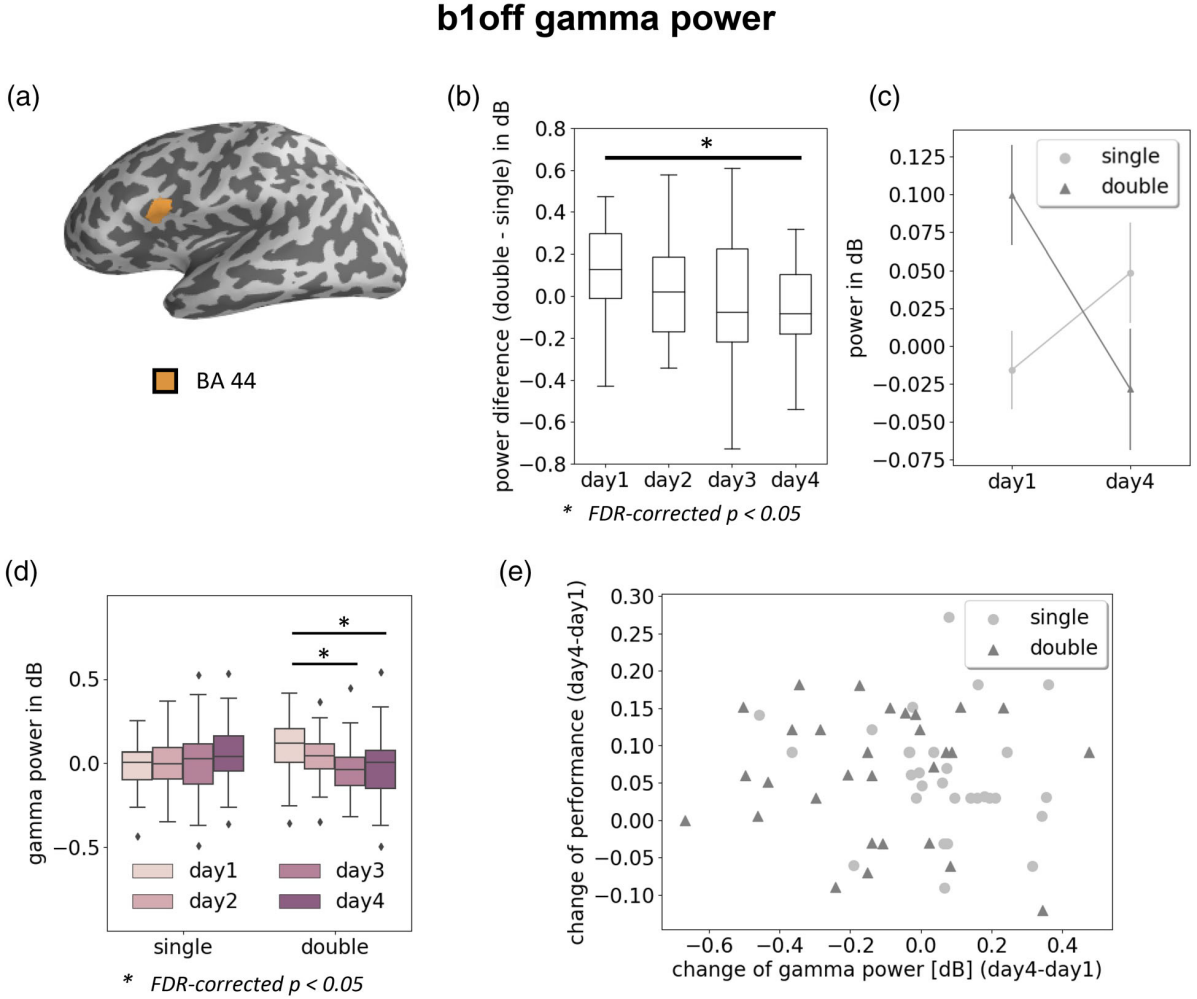
Interaction between sentence complexity and training day. (a) ROIs (orange, BA 44) showing the interaction effect on gamma power. (b) BA 44 gamma power difference between double and single embedded sentences at b1off on the four training days. On day1, the difference was significantly larger than on day4 (FDR corrected *p* < 0.05). (c) Interaction of sentence complexity and training day for gamma power in BA 44 at b1off. On day1, the gamma power of double vs. single embedded sentences was significantly higher, however, no significant difference was observed for day4. (d) Boxplot showing BA 44 gamma power of all four training days for both single and double embedded sentences. Note that gamma power decreased with training days for double but not for single embedded sentences. (e) Scatter plot of correlation between the change of performance and the change of gamma power in BA 44b at *b1off* for both single (discs) and double (triangles) embedded sentences. Changes were computed as the difference between day4 and day1 and for each participant separately

**FIGURE 5:**
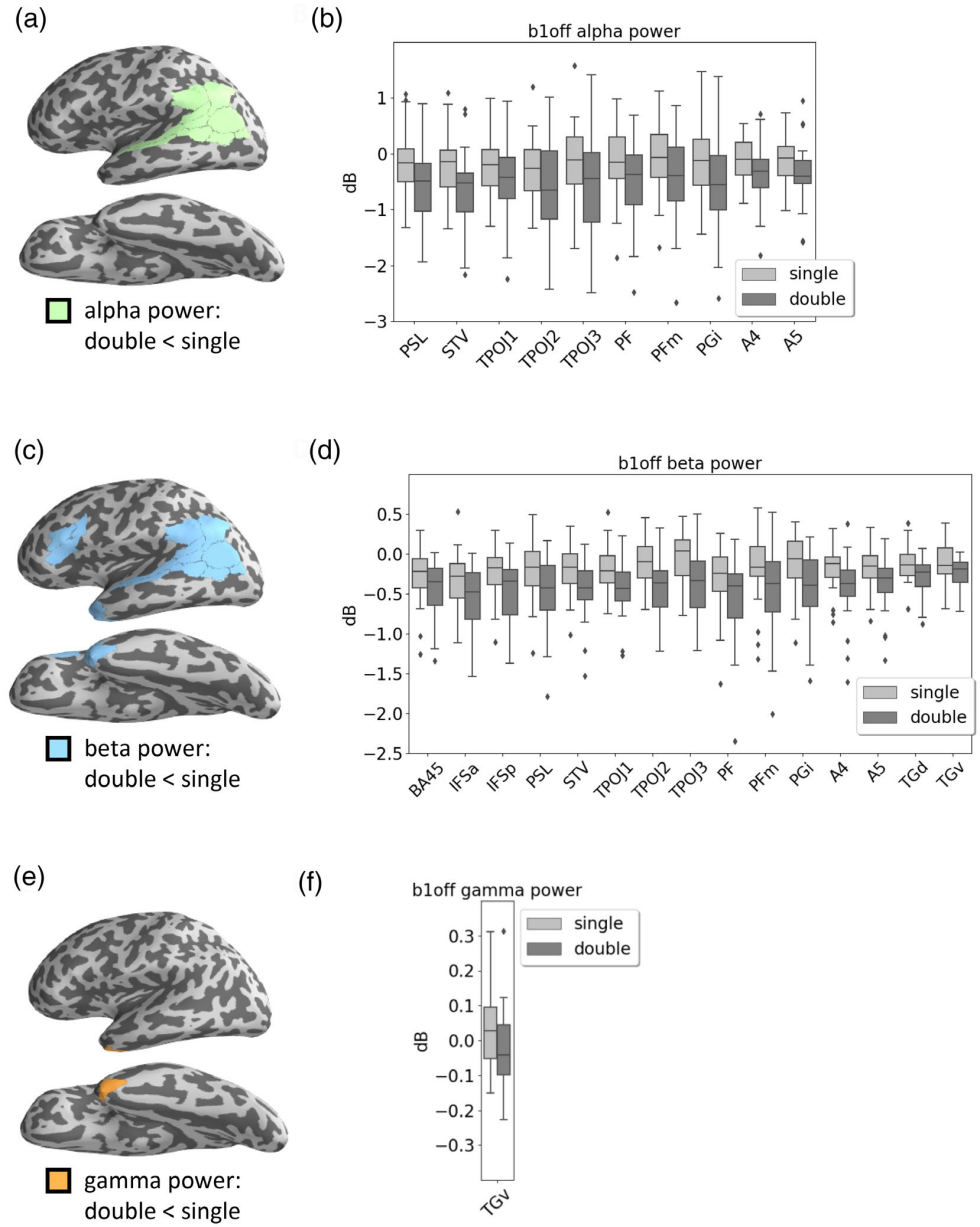
Main effect of sentence complexity at b1off. (a) The green area consists of ROIs whose alpha power significantly decreased with increasing complexity. (b) Separate alpha power of all ROIs within the green area. Alpha power differences were statistically significant in each of these ROIs. (c) The blue area consists of ROIs whose beta power significantly decreased with sentence complexity. (d) Separate beta power of all ROIs within the blue area. Beta power differences were statistically significant in each of the ROIs. (e) The orange area consists of ROIs whose gamma power significantly decreased with complexity. (f) Gamma power of all ROIs within the orange area. Gamma power differences were statistically significant in each of the ROIs. In (b), (d), and (f), ROIs were labeled according to Glasser et al. (2016)

**FIGURE 6:**
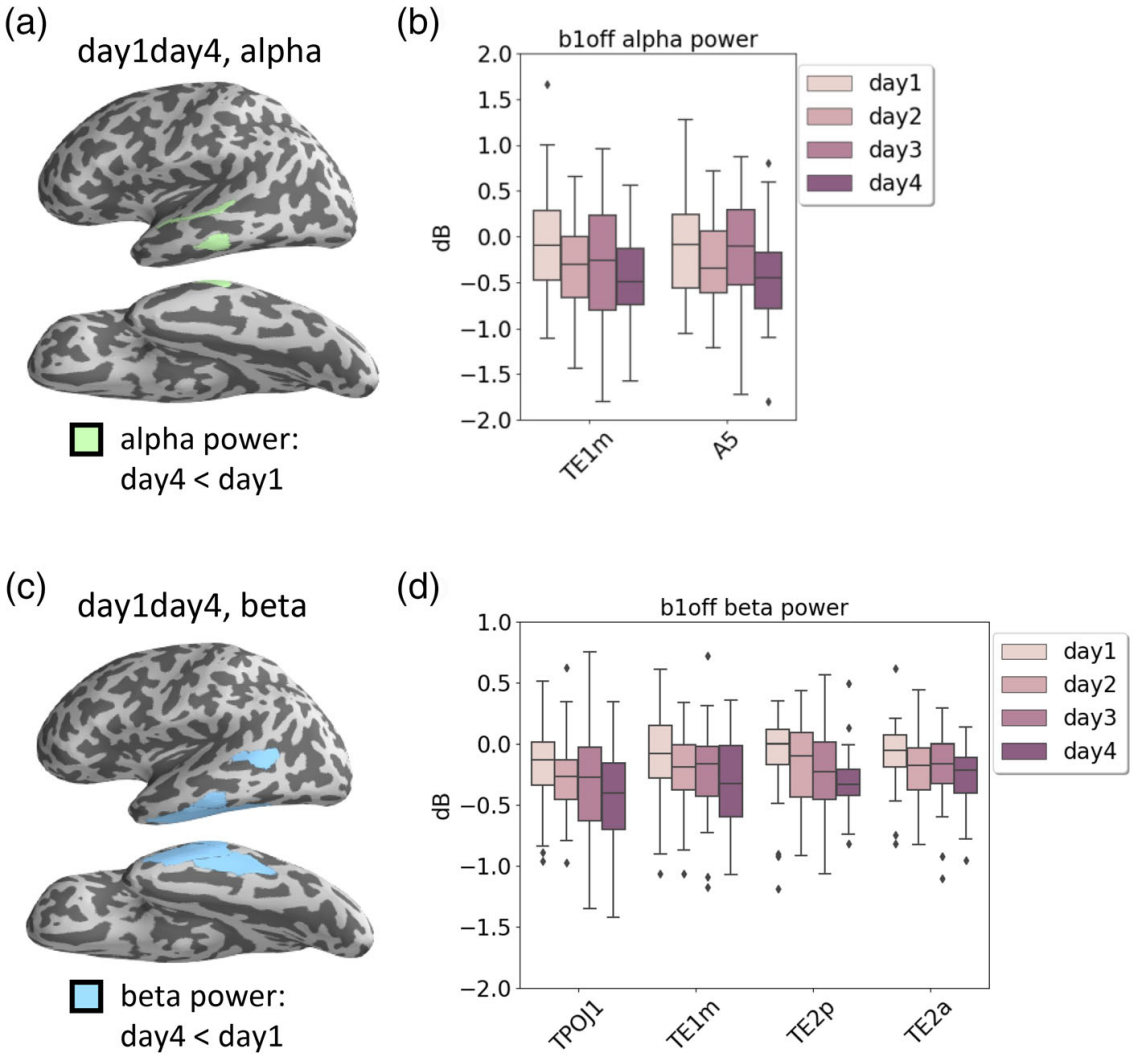
Main effect for training days. (a) The green areas comprise all ROIs showing a main effect for training days in alpha power between day1 and day4. (b) Separate alpha power of all four training days and the ROIs within the green area in A. The alpha power differed significantly between day1 and day4. (c) The blue areas comprise all ROIs showing a main effect for training days in beta power between day1 and day4. (d) Separate beta power of all four training days and the ROIs within the blue area in *C*. *beta* power differed significantly between day 1 and day 4. All ROIs were labeled according to Glasser et al., 2016

We examined the normalized power for the five power bands in the 31 ROIs at *b1off* (final closure of the relative clause of the hierarchically center embedded structures). Results of the two‐factor analysis are summarized in Figure 4 for the interaction effect, in Figure 5 for main effect of sentence complexity, and in Figure 6 for main effect of training day.

#### 3.2.1 Interaction between training day and sentence complexity

No significant interaction was found in any other band than the gamma band. A reduction of gamma power in BA 44 over training was observed for double but not for single embedding. We found a significant interaction of *sentence complexity* and *training day* for gamma power in BA 44 (orange ROI in Figure 4(a)) between day1 and day4 (Figure 4(b)). A post‐hoc test showed that on day1, gamma power of double versus single embedding was significantly higher (Wilcoxon signed‐rank test, *p* = 0.0087) (Figure 4(c)), but there was no difference on day4 (*p* = 0.14).

A further post‐hoc test of the normalized power at *b1off* of single embedding over all four training days (day1 vs. day2; day1 vs. day3; day1 vs. day4) showed no significant differences between days. However, testing the *b1off* power of the double embedding, we found that the power on day1 was higher than on day3 and day4 (*p* = 0.012 and *p* = 0.018, respectively; Figure 4(d)).

We further examined whether the individual accuracy correlated with the gamma power change in BA 44 (Figure 4(e)). We calculated the Spearman's correlation between the gamma power change and performance change for double embeddings and the training day pair: day1 and day4. No significant correlation between individual performance improvement was observed as well as a reduction of gamma power in BA 44 (*p* > 0.05).

#### 3.2.2 Main effect of sentence complexity

Testing the main effect of sentence complexity at *b1off* looks at power differences between single and double embedding, for all five frequency bands and 31 ROIs averaged over the training days. First, we found a significant decrease of both alpha and beta power for double versus single embedding in the inferior parietal cortex (PF, PFm, and PGi), temporo‐parieto‐occipital junction (PSL, STV, and TPOJ1‐3), and parts of the posterior superior temporal gyrus (A4 & A5), see Figure 5(a) and (b). Additionally, we found a significant decrease of beta power for double versus single embedded sentences in Broca's area (BA 45), the adjacent inferior frontal sulcus (IFSa & IFSp), as well as the temporal pole (TGd & TGv), see Figure 5(c), (d). Furthermore, a significant decrease of gamma power for double versus single embedding was found in the ventral part of the temporal pole (TGv), see Figure 5(e), (f ).

#### 3.2.3 Main effect of training day

A main effect of training day showed that the normalized spectral power at the final closure (*b1off*) changed over the four training days. We found significant power changes between the first and the last training day. First, we found a significant decrease in alpha power in parts of the posterior superior temporal gyrus (A5) and in parts of the middle temporal gyrus (TE1m), see Figure 6(a), (b). Second, we found a significant decrease in beta power in parts of the temporo‐parieto‐occipital junction (TPOJ1), in parts of the posterior middle temporal gyrus (TE1m), and in parts of the inferior temporal gyrus/sulcus (TE2p & TE2a), see Figure 6(c), (d).

Furthermore, we examined the correlation of these power changes with the improvement of the performance between day1 and day4 (scores averaged across single and double embedding). None of these power changes were correlated with performance improvement (Spearman's correlation test, 6 multiple tests, FDR‐corrected *p* > 0.05).

### 3.3 Correlation between performance change and functional connectivity

#### 3.3.1 Correlation between change of performance (training) and change of Granger connectivity

Between the training days 1 and 4, we observed a performance improvement as well as a decrease of gamma power in BA44, which was dependent on sentence complexity (see above). Therefore, we examined the Spearman's correlation between the performance improvements of the double embedded sentences with the Granger influence change (Δ*F*, see Section 2) for the training day1 versus day4. A significant interaction was found (performance improved and gamma power was reduced for double but not for single). Granger influence was computed between BA 44 and the 16 ROIs along the dorsal pathway, comprising parts of the IFS (IFSp, IFJa, and IFJp) adjacent to BA 44 in inferior frontal cortex, parts of the parietal cortex (PF, PFm, and PGi), and parts of the posterior temporal cortex (PSL, STV, TPOJ1, A4, A5, STSdp, STSvp, TE1m, TE1p, and PHT; see Figure 2). It has been proposed that the IFS is related to working memory functions supporting the processing of embedded structure (Makuuchi et al., 2009; Makuuchi & Friederici, 2013) and parietal cortex has been linked to semantic working memory (Gruber & von Cramon, 2003). Posterior temporal cortex is another core region for processing sentences (Friederici, 2011; Friederici, 2017a; Friederici, 2017b; Hickok & Poeppel, 2007).

**FIGURE 7:**
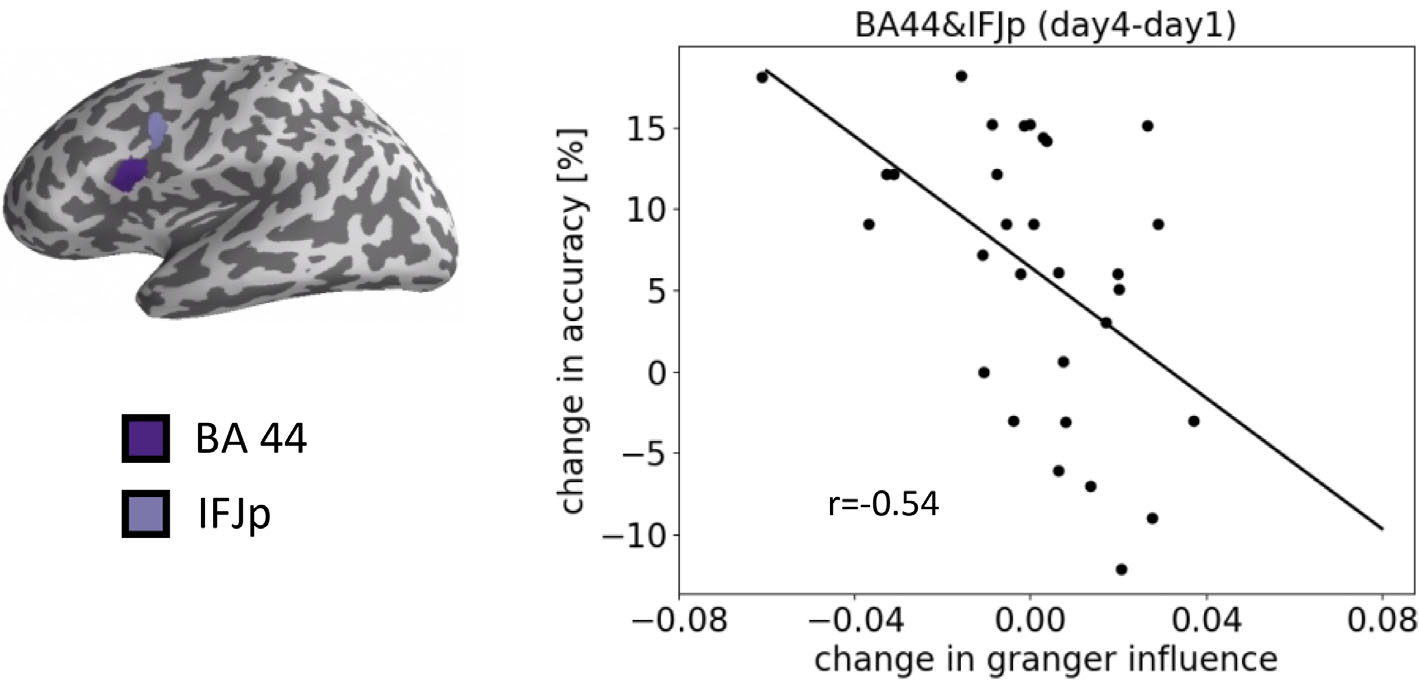
Correlation between performance and Granger influence for a pair of ROIs (BA44 & IFJp). The left panel shows the two ROIs marked in dark magenta and blue on the inflated cortical surface. The right panel shows the performance change (day 4–day 1) for the double embedded sentences plotted against the Granger influence change

We found that performance improvement was negatively correlated with Granger influence changes between BA 44 and the IFJp in the posterior part of the inferior frontal sulcus (IFS) (*r* = −0.54, FDR‐corrected *p* = 0.035; Figure 7). Negative correlation between Granger influence changes and performance improvement means that the Granger influence flow shifted towards BA 44, thus BA 44 was becoming more influenced by the other brain regions when performance improved. However, we found no such correlation for single embedding.

#### 3.3.2 Correlation between change in performance (training) and change in imaginary coherence

We computed the Spearman's correlation between the performance improvements from day 1 to day 4 for double embedding with the imaginary coherence change over the same days between BA 44 and the 16 pre‐selected ROIs related to the dorsal pathway (see Figure 2, the same ROIs are used in the previous section).

**FIGURE 8:**
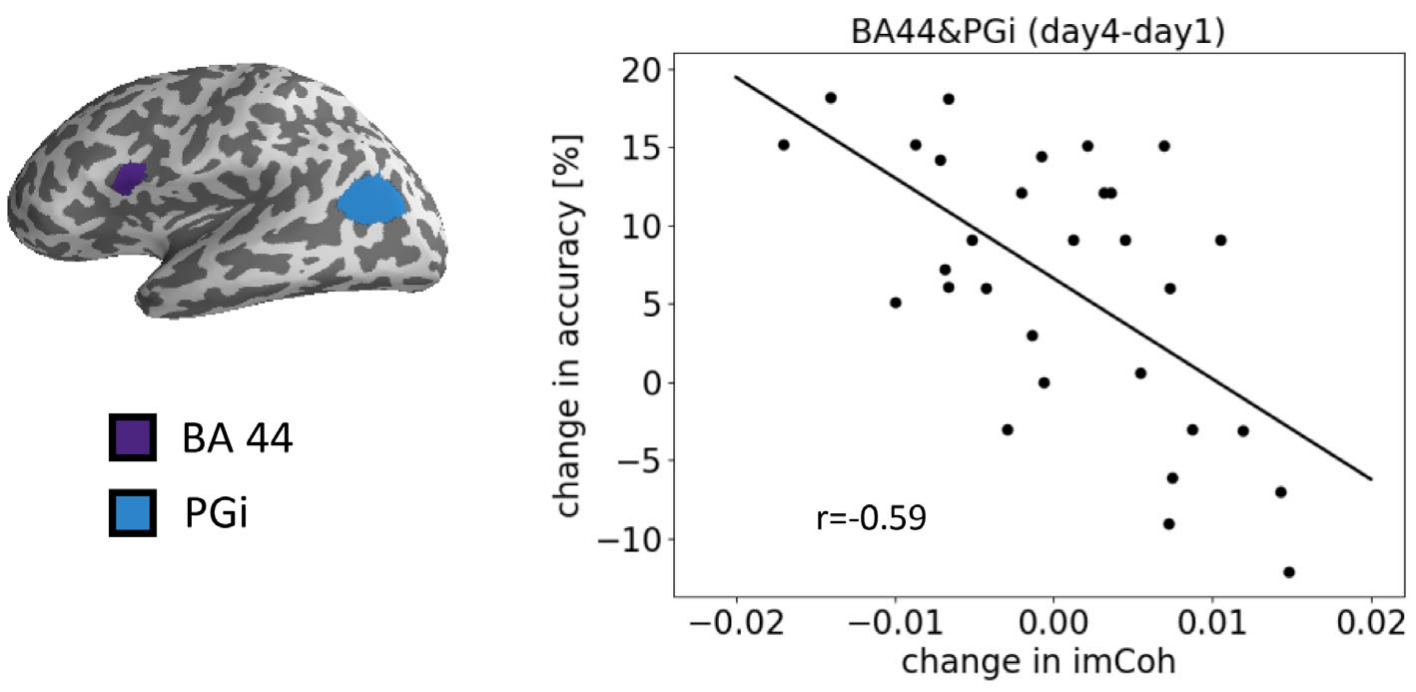
Correlation between performance and imaginary coherence between BA44 and the inferior parietal cortex. ROIs are labeled according to Glasser et al., 2016

We found that the magnitude change in the imaginary coherence between BA 44 and the PGi in the inferior parietal cortex (BA 44 phase lag to PGi) correlated with performance improvement (*r* = −0.59, FDR‐corrected *p* = 0.021, Figure 8). However, we found no such correlation for single embedding.

## 4 DISCUSSION

The present study investigated changes in behavioral performance and neural activity as reflected by MEG data in adult native German speakers. Participants underwent 4 days of intensive longitudinal training of German sentence processing with different syntactic complexities. We aimed to link the performance improvement to underlying functional changes in regions within the language network. As expected, participants' performance improved during the training period for both single and double relative clause center embedded sentences. Over the entire training period, participants made more errors for the syntactically more complex double embedded sentences. The neural basis of this performance improvement was related to changes of oscillatory activity in the fronto‐temporal language network.

With respect to brain activity, we first analyzed regional specific spectral power in different frequency bands to identify changes in *local neural synchronization* with respect to two factors: *sentence complexity* (single vs. double embedded sentences) and *training day* (day 1–day 4). We expected local synchronization in the language ROIs to change over the training period. For theta power, we did not observe any significant change, neither between sentences of different complexity nor during the course of training. For alpha and beta power, a significant reduction was observed for double as compared to single embeddings, which was independent of the training stage. This seems to reflect the higher cognitive load in the double embedding condition as these sentences were systematically longer, which raises the demands to cognitive load and working memory. Since beta power was observed to decrease with increasing cognitive load (Bastiaansen et al., 2015; Weiss & Mueller, 2012), the significant reduction of beta power for double embedding sentences compared to single ones might be explained that way. Another interpretation would be that sentences with double embeddings cause an increased level of attention when entering into the second embedding, which may lead to reduced levels of alpha and beta power at the end of the outermost (first) embedding.

An interaction between *sentence complexity* and *training day* was found exclusively for the gamma power in BA 44, such that gamma power was reduced over training only for double embedded sentences (Figure 4). In addition, main effects for both factors were identified in various ROIs (Figures 5 and 6). In the TGv (s. Figure 2, part of the anterior temporal lobe, ATL), the gamma power for single embedded sentences was higher than for double embedded sentences (Figure 5(e), (f)) and did not change over training days. This training‐independent higher gamma power for single embedded sentences in the ATL may reflect that access to the sentence's meaning is easier in syntactically less complex sentences (Fedorenko et al., 2016; Nieuwland & Martin, 2017; Rommers, Dijkstra, & Bastiaansen, 2013). Therefore, an increased gamma power might be elicited in the ATL region, which is involved in processes related to semantic combinatorics (e.g., Bemis & Pylkkänen, 2011, 2013; Matchin, Brodbeck, Hammerly, & Lau, 2019; Matchin, Hammerly, & Lau, 2017; Pallier, Devauchelle, & Dehaene, 2011; Pylkkänen, 2019). On the other hand, BA 44 is considered a core language processor for syntactic processing (e.g., Hagoort & Indefrey, 2014; Wu, Zaccarella, & Friederici, 2019; Zaccarella et al., 2017; see Friederici, 2017a, Friederici, 2017b for a systematic review). This is confirmed by our finding that activity in BA 44 varied with training for the most complex, double embedded sentences.

Our analysis was focused on the closure of the outermost embedded structure (b1off; Figure 1), where the whole embedded syntactic structure is finally complete and all syntactic processing culminates. The higher gamma power in BA 44 for the syntactically more complex sentences on the first day might represent the higher task demands (syntactic structure building and working memory) for participants when processing the rather uncommon double embedded structures (Nelson et al., 2017). During training, the participants adapted to the task and BA 44, as a central hub in the syntactic network, became better connected and more effective when building the syntactical structures. This led to a reduction of the overall neuronal recruitment, bringing the gamma power down to the level of the single embedded sentences. The increased level of gamma power with higher cognitive demands in sentence processing is in agreement with the observations of Nelson et al. (2017) and Skeide and Friederici (2016).

Because the interaction effect of local gamma band synchronization at BA 44 is very interesting, we analyzed distant neural synchronization and information flow in the gamma band between BA 44 and other areas of the language network that might cooperate with BA 44. In particular, we looked at areas belonging to the dorsal pathway proposed to support processing of sentences with complex syntax such as center embedded structures (Friederici et al., 2006; Makuuchi et al., 2009; Makuuchi & Friederici, 2013). The coupling between distant areas was studied by inspecting two different phenomena. First, we looked at the temporal development of gamma power and computed the Granger causality. We thereby established to what extent the present gamma power in one area depended on the past gamma power in another area. No phase synchronization between the areas was taken into account. Thus, we rather focused on modulatory influences between brain areas with potentially independent dynamics, which may be described as functional networks. Second, we studied coherence between the gamma band oscillations in different brain areas. Because zero‐lag correlation and zero‐phase‐lag coherence are very vulnerable to artifacts created by volume conduction (Palva & Palva, 2012), we specifically analyzed the imaginary coherence, which reflects a tight, but phase shifted, synchronization between active brain areas. This method is therefore suitable for investigating whether brain areas join together in coherent networks.

We established that the change in Granger influence from BA 44 to the posterior inferior frontal junction (IFJp) was negatively correlated to performance (Figure 7). This indicates that better training performance was associated with a change in the inflow‐outflow balance of information at BA 44, such that it is tuned to be in favor of the information flow towards BA 44. Moreover, the IFJp, as part of the inferior frontal sulcus, has been proposed as a syntactic working memory processor, also necessarily supporting the processing of embedded sentence structures (e.g., Makuuchi et al., 2009; Makuuchi & Friederici, 2013). The engagement of both regions in the same task is further corroborated by their strong mutual correlation across subjects. Therefore, these data converge on the idea that even during L1 training, processing complex syntactic structures engages not only the core syntactic processor, that is, left BA 44, but also subsidiary components such as the left IFS, which serves syntactic working memory.

Apart from these modulatory interactions between brain areas, we also found phase‐phase coupling between BA 44 and the PGi (part of IPC, inferior parietal cortex), as indicated by imaginary coherence that was correlated to training performance. This may indicate that both areas form, to some extent, an integrated network and may be engaged in the same task. The IPC has been described as part of the working memory system with special focus on verbal working memory (Awh et al., 1996; D'Esposito, Postle, Ballard, & Lease, 1999; Makuuchi et al., 2009; Makuuchi & Friederici, 2013; Meyer, Obleser, Anwander, & Friederici, 2012). Therefore, by integrating both correlation results, BA 44 might distinctively interact with different types of working memory systems (see also Makuuchi & Friederici, 2013).

Finally, it is important to note that the complex and challenging nature of the experiment inevitably limited the available statistical power. We tested 132 sentences distributed equally over the four training days, that is, we had just 33 trials per condition and training day. Hence, it is likely that the training has caused even more changes to the language network than we had the sensitivity to measure.

The experiment investigates the cognitive dynamics when processing natural speech in a setup which has an as‐large‐as‐possible lexical variance while still allowing to classify sentences into two conditions of syntactic complexity. In such a setup a large variety of brain processes may become active. When comparing the two conditions – double versus single embedded sentences – we cannot guarantee that all the other brain processes are perfectly balanced. However, the training effect was prominently detected in the left BA 44, a region previously reported as a syntactic hub by numerous neurolinguistic studies. Furthermore, since the functional connectivity between BA 44 and other areas was significantly correlated with the performance improvements, we propose that the syntactic aspect (possibly together with working memory) compose an essential part of the cognitive dynamics. To what extent other aspects less related to syntax were also relevant needs a further investigation by future studies.

In summary, we showed that L1 training of syntactically complex sentences leads to changes in neural oscillation in critical language regions, such as left BA 44, in healthy adult native speakers. Moreover, the connections between BA 44 and both the IFS and PGi might serve as a “core language region – working memory network” necessary for comprehending syntactically complex sentences. These changes are also prominently associated with the increase of behavioral L1 performance. Taken together, the current study provides novel insights for the exploration of adult functional brain plasticity even during training in one's native language.

## CONFLICT OF INTERESTS

The authors declare no conflict of interest.

## AUTHOR CONTRIBUTIONS

Jens Brauer and Thomas R. Knösche: designed the experiment and prepared the stimuli; Peng Wang and Burkhard Maess: conducted the recordings and analyzed the data; Peng Wang, Thomas R. Knösche, Luyao Chen, Angela Friederici, Burkhard Maess: discussed the results, and prepared the manuscript.

## Data Availability

Data sharing is not applicable to this article as no new data were created or analyzed in this study.
